# Prevalence and risk factors of depressive symptoms in children and adolescents with juvenile idiopathic arthritis

**DOI:** 10.1007/s00296-023-05323-4

**Published:** 2023-04-11

**Authors:** Johanna Roemer, Ariane Klein, Gerd Horneff

**Affiliations:** 1 Centre for Pediatric Rheumatology, Department of Pediatrics, Asklepios Clinic Sankt Augustin, Sankt Augustin, Germany; 2 Institute of General Medicine, University Hospital of Cologne, Cologne, Germany; 3 Department of Pediatric and Adolescents Medicine, University Hospital of Cologne, Cologne, Germany

**Keywords:** Depression, Juvenile idiopathic arthritis, JIA, Depressive disorder, Chronic disease, Childhood arthritis, Rheumatic diseases, Arthritis, Autoimmune diseases

## Abstract

Depression is a serious disorder disproportionately affecting people with chronic diseases, yet, to date is rarely recognized comorbidity in pediatric rheumatology clinical routine care. The aim of this study was to investigate the prevalence of depressive symptoms and depression in children with Juvenile idiopathic arthritis (JIA) and to identify associations to risk factors. Depressive symptoms were assessed using the Beck’s Depression Inventory (BDI)-Fast Screen Questionnaire validated for ages 13 and older and confirmed by the BDI or Hamilton Depression Scale. A cross-sectional analysis of 148 patients attending the rheumatology outpatient clinic of the Asklepios Children’s Hospital Sankt Augustin between January 2018 and May 2019 was performed. Possible associations between routinely assessed parameters of disease activity and treatment were analysed. 148 JIA patients (71.5% female), median age 14.7 years, were included. The prevalence for depressive symptoms was 13% and for depression 9.5%, of which 71.4% were newly identified with depression. Significant associations with depressive symptoms included rheumatoid factor negative polyarthritis, higher pain scores, functional limitations, higher disease activity, decreased general well-being, higher number of medications taken and not being in remission. In addition, poor treatment response (persistent pain despite therapy) and failure to achieve minimal activity/remission of disease despite intensified therapy with biologics correlated significantly with depressive symptoms. Depressive symptoms are an important comorbidity in JIA. Early recognition and treatment of psychological distress is essential to prevent deterioration in quality of life and long-term prognosis. Consequently, treat-to-target principles should include mental health as a therapeutic goal.

## Introduction

Depression is one of the most common diseases worldwide. The prevalence of depression has increased dramatically in recent decades and accounts for a large proportion of the global burden of disease and disability-adjusted life years [1]. In comparison with other chronic conditions, depression alone has the greatest impact on overall health, and as a comorbidity, it deteriorates health even more [2]. Depressive symptoms in children and adolescents lead to a significantly reduced quality of life, functional limitations in daily life, social contacts and leisure activities, lowers school performance, and jeopardizes child development [3–8].

Depressive disorders in childhood and adolescence have a prevalence of 4–5% worldwide. In a German analysis, 8.2% of adolescents between the ages of 12 and 17 exhibit depressive symptoms [9, 10]. From adolescence onward, girls are more frequently affected than boys [11]. The symptoms focus on sadness, loss of joy and interests, loss of self-esteem, social withdrawal, concentration problems, changes in appetite, and sleep disturbances. Moderate and severe forms are accompanied by suicidal thoughts and actions [12]. Onset varies widely, but averages around age 14 [13].

The bidirectional relationship between inflammation and depression is the subject of current research and is of particular interest considering patients with chronic inflammatory diseases. Children and adolescents with chronic diseases are significantly more likely to suffer from depression than the general population [14, 15]. Earlier studies on depression in JIA indicate a prevalence between 7 and 36% [16]. Of note, juvenile idiopathic arthritis (JIA) patients have been found to have increased enzyme activity in dopamine and serotonin metabolism, which may explain a tendency to develop depression, anxiety disorders and cognitive impairment [17]. Another study examined cerebral blood flow in JIA patients and found similarities to the blood flow pattern in patients diagnosed with depression and anxiety disorder [18].

It has been postulated that the increased prevalence of depression in patients with chronic illnesses is related to special burdens, such as chronic pain, functional limitations, medication use, side effects of medications, restriction of leisure time, and, depending on the illness, frequent examinations and painful blood draws [19, 20]. In addition to the burdens caused by chronic illness, depression in turn limits everyday functioning. In patients with JIA, for example, higher depression scores correlated with poorer school performance and truancy in studies [6].

Overall, it appears to be of great importance to systematically screen and treat patients with inflammatory diseases for the presence of depression [2]. However, there is a lack of reliable data from large study populations to estimate the prevalence and disease-related factors that influence the development of depression to provide early and effective help to JIA patients at highest risk. The aim of this study was to investigate the prevalence of depressive symptoms and depression in children with Juvenile idiopathic arthritis (JIA) and to identify associations to risk factors.

## Methods

A cross-sectional analysis of 148 patients attending our rheumatology outpatient clinic between January 2018 and May 2019 was performed. Possible associations between depression questionnaire results and routinely assessed parameters of disease activity and treatment were analysed. The study cohort consisted of already diagnosed JIA patients as classified by ILAR over the age of 13 years visiting the outpatient care unit for their routine consultations between January 2018 and May 2019. Diagnoses included all JIA categories. There were no exclusion criteria apart from age and diagnosis. After giving verbal consent (self-determined or in case of younger children additional parental consent) the patients were handed the Beck Depression Inventory-Fast Screen (BDI-FS) to identify depressive symptoms. The BDI-FS is a short self-assessment questionnaire with seven questions about depressive symptoms within the last 2 weeks. It measures the severity of depression with seven items relating to mood (sadness, loss of pleasure), cognitive components of depression (pessimism, feelings of failure, self-rejection), and suicidal ideation. The BDI-FS is validated for patients aged 13 years and older [21]. It can be completed and evaluated in less than 5 min, making it well suited for screening as part of a routine outpatient paediatric rheumatology examination [21].

A sum score between 0 and 21 can be obtained, with between 0 and 3 points for each item. A score ≥ 4 points is considered abnormal (4–8 = mild, 9–12 = moderate, 13–21 = severe), with special attention to the question about suicidal thoughts. The sensitivity of the BDI-FS is reported to be 0.86 and the specificity 0.93 [22]. In addition, several studies confirmed a strong correlation of Beck Depression Inventory-second edition (BDI-II) and BDI-FS (*r* = 0.85 to *r* = 0.92) [23, 24].

The BDI-FS questionnaire was evaluated immediately during the consultation. If patients demonstrated an abnormal score ≥ 4 points or indications of suicidality, a psychological exploratory interview was conducted by two psychologists in the rheumatology outpatient clinic. In consultation with the patient and parents the interview was conducted, if possible on the same day, to verify the presence of a possible depressive disorder or suicidality and to plan further procedure. The psychologists assessed depressive symptoms with comprehensive tests: either the Beck Depression Inventory (BDI) or the Hamilton Depression Scale. If, after examination and assessment the child psychologists confirmed the suspicion of a psychological disorder, a referral to a child and adolescent psychiatric facility was made outside of the present study.

In addition to the questionnaire results, we used the following data collected for the time period between January 2018 and May 2019 for retrospective analysis: patients’ age, gender, rheumatologic main diagnosis, concomitant diseases (up to three additional), age at onset of disease, disease duration, disease activity with juvenile arthritis disease activity (JADAS10), JADAS10 remission defined as JADAS10 < 1, JADAS10 minimal disease activity (MDA, defined as JADAS10 1.1–3.8 for polyarticular course; 1–2 for oligoarticular course, JADAS10 moderate and high disease activity defined as JADAS10 > 3.8 (polyarthritis)/ > 2 (oligoarthritis) [25], inflammatory parameters erythrocyte sedimentation rate (ESR) and C-reactive protein (CRP), number of active joints (joints affected by active arthritis, defined as swollen and/or simultaneously painful with pressure or movement with a limited range of motion; joint count performed by consulted physician in our outpatient clinic), number of painful joints, number of joints with limited range of motion, physician global assessment of disease activity (10 cm visual analogue scale (VAS): 1 = good, 10 = poor), global evaluation of the general well-being by the patient (10 cm VAS: 1 = good, 10 = bad), pain assessment (10 cm VAS: 1 = good, 100 = poor), functional burden of disease childhood health assessment questionnaire (CHAQ-DI) as functional score recording the limitation of the quality of life in children with JIA, pharmacomedical therapy (disease modifying antirheumatic drugs (DMARDs), biologics, prior therapies, concomitant medications, non-steroidal anti-inflammatory drugs (NSAIDs) and whether patients were already undergoing psychotherapy.

### Statistical analyses

The pseudonymized data were merged into a “Microsoft Access (Microsoft, Redmond, WA, USA)” data set. Analysis was performed using “Microsoft Excel (Microsoft, Redmond, WA, USA)” and “IBM SPSS Version 25 (IBM, Armonk, NY, USA)” for statistical analysis. Continuous variables were tested for normal distribution using the Kolmogorov–Smirnova test and bar charts. Descriptive statistics were reported as medians and interquartile ranges for continuous variables and frequencies for categorical variables. The variables were subsequently tested for correlations with the three binary outcomes (1) abnormal BDI, (2) depression, (3) newly diagnosed depression using tests for non-normally distributed data. For metric variables, the Mann–Whitney *U* test was used, and for categorical variables, the chi-square test. The Kruskal–Wallis test was used to evaluate the individual responses in the BDI-FS questionnaire. Reported *p* values ≤ 0.05 were considered statistically significant.

## Results

### Patient characteristics

A total of 148 JIA patients [42 (28%) male; 106 (72%) female] were included in the study. The median age of patients was 14.6 (13, 16) years with a minimum of 13 and a maximum of 18 years. The median (IQR) age at JIA onset was 10 (7, 12) with a disease duration of 4 (2, 7) years. The most common JIA categories among the patients surveyed were RF-negative polyarthritis (*n* = 44, 29.7%), persistent oligoarthritis (*n* = 29, 19.6%), extended oligoarthritis (*n* = 25, 16.9%), and enthesitis-associated arthritis (*n* = 24, 16.2%) (Table 1).

**Table 1:** Association of patients’ characteristics, juvenile idiopathic arthritis (JIA) categories and disease activity markers according to Beck’s Depression Inventory Fast Screen (BDI-FS) scores and diagnosis of depression in 148 JIA patients

	BDI-FS < 4 (*n* = 129, 87%)	BDI-FS ≥ 4 (*n* = 19, 13%)	*p*	No depression (*n* = 134, 90.5%)	Depression (*n* = 14, 9.5%)	*p*
Female (*n* = 106, 71.6%)	89 (69%)	17 (89.5%)	0.06	93 (69.4%)	13 (92.9%)	0.06
Age, years	14.6 (12.8, 16.1)	15.5 (14.2, 16.6)	0.06	14.7 (12.83, 16.2)	14.64 (13.83, 16.67)	0.32
Age at disease onset, years	9 (6, 12)	11 (9, 12.25)	0.06	10 (6, 12)	11 (8.5, 12.75)	0.14
Disease duration, years	4 (2, 7)	2.5 (1.75, 6.25)	0.39	4 (2, 7.5)	2 (1.25, 5.75)	0.23
JIA categories						
SJIA (*n* = 4, 2.7%)	4 (3.1%)	0 (0%)	0.436	3 (2.2%)	1 (7.1%)	0.282
RF- polyarthritis (*n* = 44, 29.7%)	34 (26.4%)	10 (52.6%)	0.019	35 (26.1%)	9 (64.3%)	0.003
RF + polyarthritis (*n* = 3, 2%)	1 (0.8%)	2 (10.5%)	0.005	2 (1.5%)	1 (7.1%)	0.153
Persistent oligo (*n* = 29, 19.6%)	28 (21.7%)	1 (5.3%)	0.092	27 (20.1%)	2 (14.3%)	0.599
Extended oligo (*n* = 25, 16.9%)	24 (18.6%)	1 (5.3%)	0.147	25 (18.7%)	0 (0%)	0.076
Enthesitis-associated arthritis (*n* = 24, 16.2%)	22 (17.1%)	2 (10.5%)	0.471	24 (17.9%)	0 (0%)	0.084
Psoriatic arthritis (*n* = 16, 10.8%)	13 (10.1%)	3 (15.8%)	0.454	16 (11.9%)	0 (0%)	0.171
Unclass JIA (*n* = 2, 1.4%)	2 (1.6%)	0 (0%)	0.585	2 (1.5%)	0 (0%)	0.645
Disease activity markers						
JADAS10	2 (0, 5.15)	5.3 (1.85, 10.25)	0.008	2 (0, 5.85)	4 (0.5, 8.5)	0.213
JADAS-acceptable disease activity	16 (13.2%)	3 (17.6%)	0.620	16 (12.8%)	3 (23.1%)	0.306
JADAS-minimal disease activity	28 (23.1%)	4 (23.5%)	0.972	29 (23.2%)	3 (23.1%)	0.992
JADAS-remission	48 (39.7%)	2 (11.8%)	0.025	47 (37.6%)	3 (23.1%)	0.3
Active joints	0 (0, 1)	0.5 (0, 2.5)	0.179	0 (0, 1)	0 (0, 3)	0.34
Physician global disease activity 0–10	0 (0, 1)	1 (0.08, 2.88)	0.026	0 (0, 1)	0.5 (0, 2.25)	0.248
CHAQ-DI	0 (0, 0.375)	0.44 (0.19, 0.81)	< 0.001	0 (0, 0.375)	0.375 (0, 0.69)	0.026
Global well-being 0–10	0.7 (0, 2.5)	3.5 (1.1, 5.8)	0.002	1 (0, 3)	2 (0.9, 4.75)	0.119
Tender joints	1 (0, 2)	2 (0.5, 4)	0.017	1 (0, 2)	2 (1, 3.5)	0.034
Pain 0–10	1 (0, 4)	4 (1.3, 5.5)	0.002	1 (0, 4)	3 (1.65, 4.75)	0.059
Pain despite analgesics/NSAIDs	43 (75.4%)	13 (100%)	0.046	47 (79.7%)	9 (81.8%)	0.87
CRP	0.25 (0.08, 1.45)	0.72 (0.07, 3.49)	0.466	0.33 (0.09, 1.8)	0.1 (0.04, 1.26)	0.162
ESR	8 (4, 15.5)	13 (5.5, 19.5)	0.302	8 (4, 17)	9 (5.5, 14)	0.964
Treatment						
Antirheumatic drugs^a^	0 (0, 1)	2 (1, 3)	0.026	1 (1, 2)	2 (1.75, 3)	0.016
Methotrexate (*n* = 48, 32%)	41 (31.8%)	7 (36.8%)	0.66	43 (32.1%)	5 (35.7%)	0.783
Sulfasalzine (*n* = 5, 3%)	4 (3.1%)	1 (5.3%)	0.626	5 (3.7%)	0 (0%)	0.462
Adalimumab (*n* = 17, 12%)	13 (10.1%)	4 (21.1%)	0.161	15 (11.2%)	2 (14.3%)	0.73
Etanercept (*n* = 27, 18%)	24 (18.6%)	3 (15.8%)	0.767	25 (18.7%)	2 (14.3%)	0.687
Tocilizumab (*n* = 16, 11%)	14 (10.9%)	2 (10.5%)	0.966	13 (9.7%)	3 (21.4%)	0.179
All TNFi (*n* = 49, 33%)	40 (31%)	9 (47.4%)	0.157	44 (32.8%)	5 (35.7%)	0.828
No drugs (*n* = 22, 15%)	19 (14.7%)	3 (15.8%)	0.903	20 (14.9%)	2 (14.3%)	0.949

Results in table presented as median (IQR1, IQR3) and *n* (%)

*ESR* erythrocyte sedimentation rate, *CRP* C-reactive protein, *CHAQ-DI* Childhood Health Assessment Questionnaire Disability Index, *JADAS10* 10-joint juvenile arthritis disease activity score, *SJIA* systemic juvenile idiopathic arthritis, *RF* + *Polyarthritis* rheumatoid factor negative polyarthritis, *RF- Polyarthritis* rheumatoid factor positive polyarthritis, Persistent Oligo: persistent Oligoarthritis, *Extended Oligo* extended Oligoarthritis

^a^Including *csDMARD* conventional synthetic disease modifying antirheumatic drug, *bDMARD* biological disease modifying antirheumatic drug), steroids and *NSAID* non-steroidal anti-inflammatory drugs, *BDI-FS* Beck depression inventory-fast screen, *TNFi* tumor necrosis factor inhibitor

### Prevalence of depressive symptoms and depression

More than 1 in 8 patients (13%) with JIA presented with depressive symptoms (BDI-FS score ≥ 4), 9.5% suffered from clinically diagnosed depression and 6% even had suicidal ideation. Regardless of an abnormal total score ≥ 4 points, some statements in the BDI-FS occurred particularly frequently. One in three patients (29%) with JIA reported a loss of joy, and one in five (20.9%) reported sadness. Self-reproach (17.6%) and despondency (15.5%) were also common. The most prevalent comorbidities were pain amplification syndrome (*n* = 7, 4.7%), anxiety disorders (*n* = 3, 2%), and self-harming behaviour (*n* = 2, 1.4%). 5.4% of patients were undergoing psychotherapeutic treatment (including treatment for reasons other than depression) at the time of the survey, and 2.7% were being treated with antidepressants. Of the 9.5% of patients with clinical depression, 71.4% (10 patients) were newly diagnosed with depression.

### Risk factors for depressive symptoms and depression in children and youth with JIA

Significantly correlating influencing factors for depressive symptoms and depression were seronegative/seropositive polyarthritis, pain, functional limitations, higher number of painful joints, higher physician’s global assessment of disease activity, higher patient’s assessment of limitation of general well-being, higher disease activity (median JADAS10), non-achievement of JADAS10 minimal disease activity or remission, NSAID use, higher number of medications taken, higher number of concomitant medications, psychiatric comorbidities, and concomitant psychotherapy (Table 1).

No statistically significant correlations were found for age, gender, JIA category (except polyarthritis), disease duration, CRP/ESR, treatment with conventional synthetic disease modifying antirheumatic drug (csDMARD)/biologics, glucocorticoid intake, number of previous therapies, number of active/movement-impaired joints, therapy duration, frequency of treatment application and concomitant somatic diseases (Table 1).

**Fig. 1:**
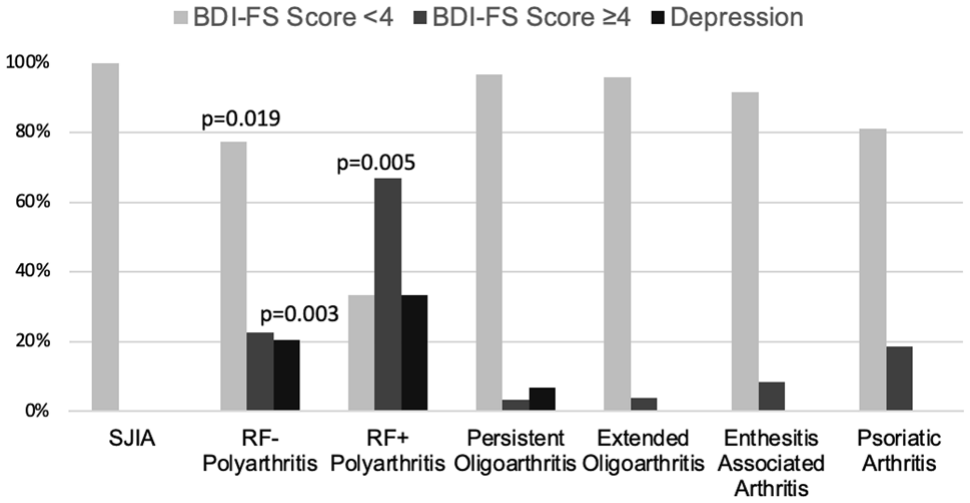
Prevalence of Beck Depression Inventory-Fast Screen (BDI-FS) score value and depression comparing JIA categories. SJIA (systemic juvenile idiopathic arthritis), RF- Polyarthritis (RF-negative polyarthritis), RF + Polyarthritis (RF-positive polyarthritis)

**Fig. 2:**
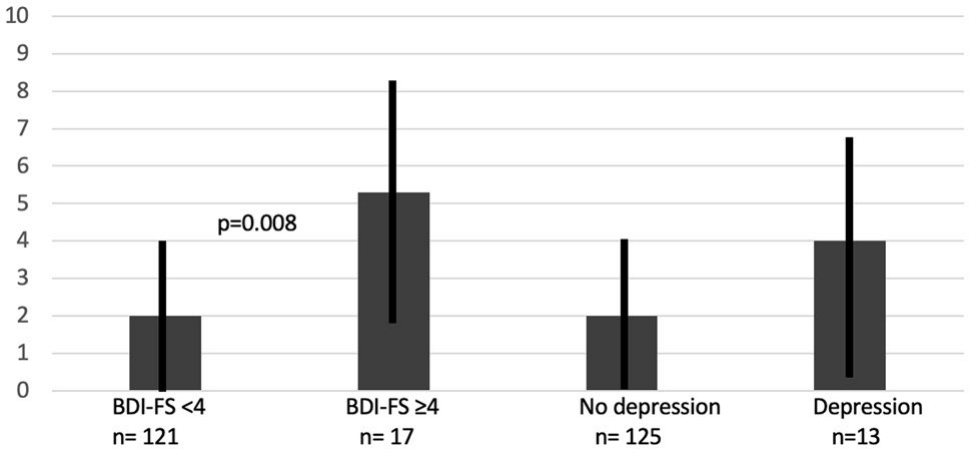
Beck Depression Inventory-Fast Screen (BDI-FS) score/depression and disease activity (JADAS10; median, IQR). JADAS10 (10-joint juvenile arthritis disease activity score)

**Fig. 3:**
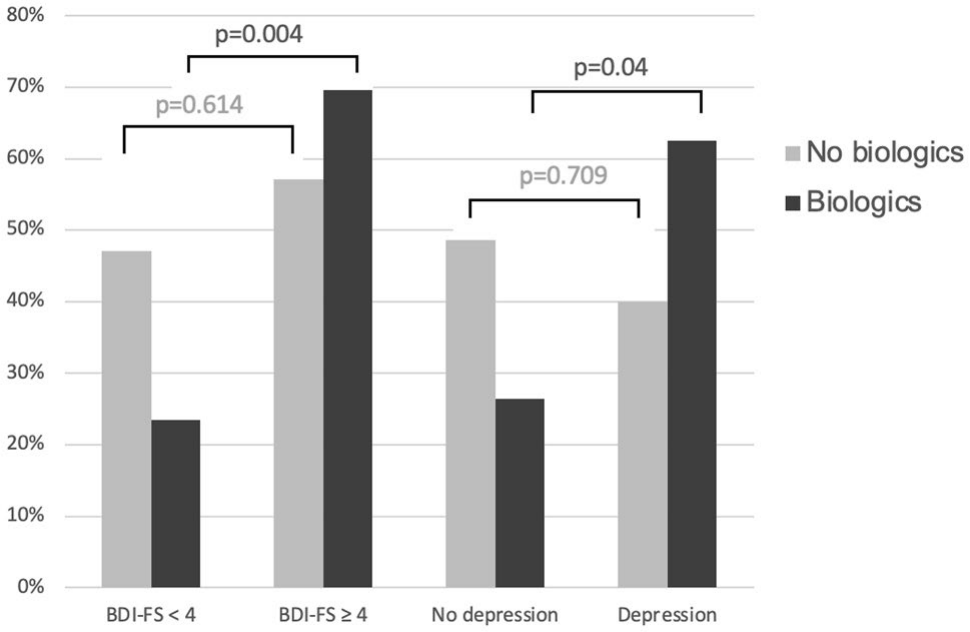
Beck Depression Inventory-Fast Screen (BDI-FS) score/depression in patients with JADAS10 moderate/high disease activity (JADAS10 > 3.8 (polyarthritis)/ > 2 (oligoarthritis) in relation to therapy with biologics. JADAS10 (10-joint juvenile arthritis disease activity score), BDI-FS (Beck Depression Inventory-Fast Screen)

**Fig. 4:**
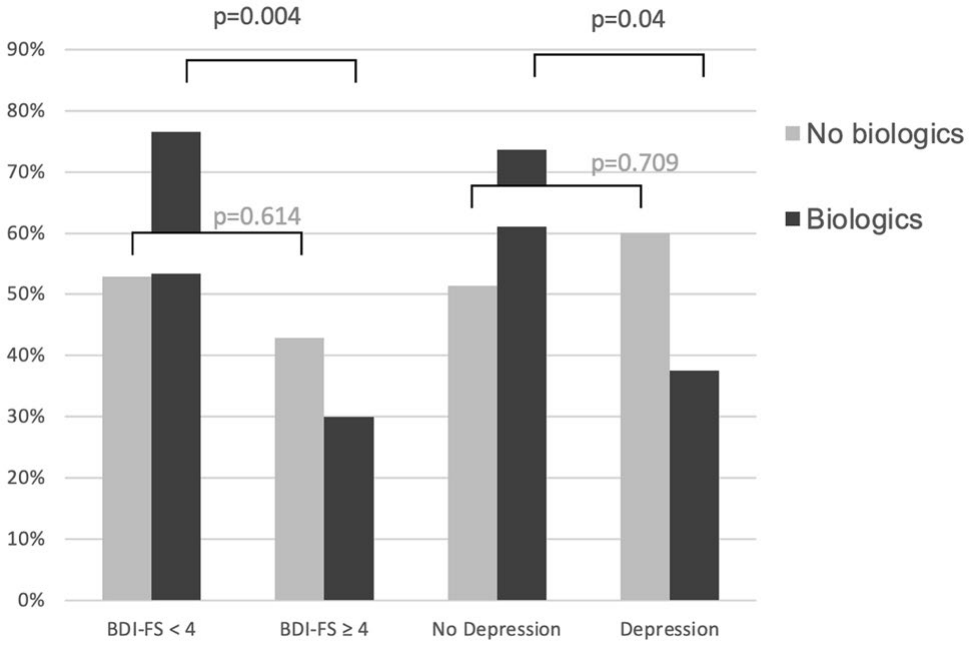
Beck Depression Inventory-Fast Screen (BDI-FS) score/depression in patients with JADAS10 minimal disease activity/remission (JADAS10 < 3.8 polyarthritis; < 2 oligoarthritis) with and without therapy with biologics. JADAS10 (10-joint juvenile arthritis disease activity score), BDI-FS (Beck Depression Inventory-Fast Screen)

Interestingly, JIA patients with poor treatment response despite intensified treatment as indicated by the use of biologics were particularly affected by higher BDI-FS scores and depressive symptoms. This corelation was highly significant if patients did not achieve disease remission (*p* = 0.004) or minimal disease activity while receiving intensified therapy with biologics (*p* = 0.04). In addition, median JADAS10, CHAQ-DI and pain under biologic therapy also correlated with depressive symptoms, whereas this was not the case in patients who did not receive biologics as indicator for an intensified antirheumatic treatment (Table 2 and Figs. 1, 2, 3, 4).

**Table 2:** Disease activity parameters according to treatment with/without biologics and Beck’s Depression Inventory Fast Screen (BDI-FS) scores/diagnosis of depression

JIA patients receiving biologics (*n* = 65, 44%)						
	BDI-FS < 4	BDI-FS ≥ 4	*p*	No depression	Depression	*p*
JADAS10	0.5 (0, 3)	6.5 (1.9, 9.6)	< 0.001	0.6 (0, 4.4)	5 (1, 8.8)	0.022
JADAS moderate/high disease activity	23.5%	7%	0.004	14 (26.4%)	5 (62.5%)	0.04
JADAS MDA/remission	76.5%	30%	0.004	39 (73.6%)	3 (37.5%)	0.04
Pain	59.3%	100%	0.009	36 (63.2%)	7 (87.5%)	0.173
No pain	40.7%	0%	0.009	21 (36.8%)	1 (12.5%)	0.173
Pain VAS 0–10	0.5 (0, 2)	4.3 (3, 5.75)	< 0.001	0.5 (0, 3)	4 (3, 4.9)	0.012
CHAQ-DI	0 (0, 0.2)	0.5 (0.25, 1)	< 0.001	0 (0, 0.375)	0.5 (0.28, 0.94)	< 0.001

JIA patients not receiving biologics (*n* = 83, 56%)						
	BDI-FS < 4	BDI-FS ≥ 4	*p*	No depression	Depression	*p*
JADAS10	4 (1, 9)	11 (7.5, –)	0.239	4 (1, 9)	8 (2, –)	0.570
JADAS moderate/high disease activity	47.1%	57.1%	0.614	48.6%	2 (40%)	0.709
JADAS MDA/remission	52.9%	42.9%	0.614	51.4%	60%	0.709
Pain	72%	100%	0.083	74%	83.3%	0.613
No pain	28%	0%	0.083	26%	16.7%	0.613
Pain VAS 0–10	2 (0, 4)	1.5 (0.5, 6)	0.398	2 (0, 4)	2.2 (0.6, 4.5)	0.961
CHAQ-DI	0 (0, 0.375)	0.375 (0, 0.625)	0.086	0 (0, 0.375)	0 (0, 0.31)	0.474

Data set was not complete for all parameters. The number of available parameters was JADAS *n* = 61/77, pain score *n* = 43/62, CHAQ *n* = 63/80. Results in table presented as median (IQR1, IQR3) and *n* (%)

*bDMARD/biologics* biological disease modifying antirheumatic drug, *BDI-FS* Beck depression inventory-fast screen, *CHAQ-DI* Childhood Health Assessment Questionnaire Disability Index, *JADAS10* 10-joint juvenile arthritis disease activity score, *MDA* minimal disease activity

Patients who reported pain despite analgesic therapy also showed a significant correlation with abnormality in the BDI-FS (*p* = 0.046) (Table 2).

## Discussion

In the total cohort, more than 1 in 8 patients (13%) presented with depressive symptoms with a BDI-FS score ≥ 4 points, and nearly 1 in 10 patients (9.5%) suffered from clinical depression. 6% reported suicidal ideation. However, only 5.4% of the patients were undergoing psychotherapeutic treatment (including treatment for other reasons than depression) at the time of the survey, and 2.7% were receiving medication with antidepressants. As expected, depressive disorders increased with age, especially in girls [26].

The prevalence of depression in children and adolescents in Germany is reported to range from 3 to 10%, depending on the study [27–29]. With a prevalence of 13% for depressive symptoms and 9.5% for clinical depression, the study cohort is affected by depression at least as frequently, if not more frequently. Most patients (71.4%) were newly diagnosed with depression within this study. This highlights the large discrepancy between the need for treatment and current diagnostics regarding depression.

The exact prevalence of depressive disorders in JIA patients remains unclear as previous studies on depression in JIA patients reported clinically significant depressive symptoms with a prevalence varying from 7 to 36% [5, 7, 16, 23, 30–33]. The variance in prevalence can be explained by the heterogeneity of the study populations in terms of JIA categories, different inclusion criteria, and the use of different validated screening questionnaires [16]. Previous studies which did not find clinically significant depressive symptoms all used the Children’s Depression Inventory (CDI) or Beck Depression Inventory (BDI), which may be explained by the fact that in the studies without clinically significant depressive symptoms, acutely ill patients and those with a known mental comorbidity were excluded a priori [3, 16, 34].

In contrast to previous studies, we included patients with pre-existing mental comorbidities in our analysis. The failure to include them in previous studies may have led to an underestimation of the prevalence of depression. Conversely, the inclusion of other mental comorbidities carries the risk of inexact differentiation between risk factors that are specific to depression and those that may be attributed to other mental comorbidities [16]. However, considering that depression and other psychological illnesses frequently go hand in hand, risk factors might tend to overlap. Follow-up studies with larger cohort numbers and thus higher numbers of the various mental comorbidities would, therefore, be interesting for a subgroup analysis.

Another explanation for the heterogeneity of data on the prevalence of depressive symptoms and depression may lie in the different ways data were obtained. Frequently, parents were interviewed rather than the children and adolescents themselves [26]. However, several studies found a large discrepancy between parents’ statements and children’s and adolescents’ self-assessments [26, 34].

We found that depressive symptoms were more common in JIA patients with seronegative and seropositive polyarthritis. Patients with RF-negative polyarthritis in particular were significantly more likely to experience depression. This result compares well with findings from a few earlier studies that showed that patients with polyarticular course are at higher risk for depression compared to other categories [16, 23]. In the study by Hanns et al., patients with polyarticular course showed increased depressive symptoms compared to patients with oligoarthritis and ERA–JIA [23].

Similar to most previous studies, we found that depressive symptoms were associated with the global patient assessment, pain and physical functional impairment as measured by CHAQ-DI [16]. Hanns et al. even showed that a higher depression baseline predicted increased functional impairment and pain 1–4 years later [23]. Notably, few previous studies did not find this correlation, likely due to the study limitations of earlier studies mentioned above [16, 35]. In contrast to some earlier studies, we could not find a correlation between the number of movement-restricted joints and “active joint count” and depressive symptoms [16]. This may be due to the fact that in our study the number of active and movement restricted joints was extremely low, making it hard to examine correlations; a finding that, on the whole, highlights the strides that the treatment of JIA has made.

Disease activity and refractory disease were also of particular interest in our analysis. In the total JIA group, the median JADAS10 as a parameter for assessing disease activity was 2 (0, 6). Higher JADAS values correlated with the occurrence of depressive symptoms as measured by BDI-FS scores. 36.2% of JIA patients were in remission of their disease according to JADAS10. Patients with depressive symptoms (abnormal BDI-FS) were less likely to be in remission. This association was statistically significant.

Patients in this study with active disease or higher disease activity showed depressive symptoms more frequently. This observation is in accordance with other studies such as that by Hanns et al. which reported that increased disease activity in the first year correlated particularly significantly with depressive symptoms [23]. A study by Barth et al. that only included patients over 18 years of age and only distinguished between active and inactive disease, also found a significant association between active disease and depression [24]. They concluded that active JIA in adulthood also meant longer disease duration and a more severe course of the disease, which in turn could account for depressive symptoms.

Until now, associations between depressive symptoms and medication in JIA patients have rarely been examined [16, 26]. This is particularly interesting in light of current research on the use of immunomodulatory drugs in the treatment of depression. Consistent with previous studies our analysis could not detect an association between treatment with either csDMARD or biologics and presence of depression or depressive symptoms.

Yet interestingly, JIA patients who did not achieve remission or minimal disease activity (MDA) according to JADAS10 despite intensified therapy (for which a biologic is used) were significantly more likely to experience depressive symptoms and depression. The highest rates of abnormal BDI-FS (70%) were observed in JIA patients with moderate/high disease activity despite treatment with biologics. In contrast, patients who reached minimal disease activity or remission while on biologics had a low prevalence of abnormal BDI-FS (30%). The median score of JADAS10 also correlated significantly with depressive symptoms in patients on biologic therapy. There was no association with depressive symptoms in patients not achieving JADAS10 remission or MDA, who were not treated with biologics. The association between higher JADAS10 levels and depressive symptoms was also evident for patients receiving csDMARD therapy. Here, however, the association remained without statistical significance due to the small number of cases. Patients without immunomodulatory therapy had the lowest JADAS10 values and were already in remission or MDA. There was no association with depressive symptoms.

Thus, persistence of active disease or higher disease activity despite intensive antirheumatic therapy appears to be associated with depressive symptomatology and depression, and that adjustment of therapy at higher disease activity is not only necessary for physical but also for mental health.

### Strengths and limitations

A total of 148 JIA patients were included in this retrospective analysis, making this study cohort one of a few with such an extensive cohort size and provides new insights on risk factors for adolescent JIA patients [16]. In addition, it presents relevant results and new insights for treatment and prevention of depressive disorders in patients with JIA. Although younger children are far more frequently affected by JIA, only children and adolescents aged > 13 years could be included in the study, as the BDI-FS is only validated from this age upwards. Because of the retrospective evaluation, the inclusion criteria were deliberately defined broadly to include as much available patient data as possible which may have diluted findings for specific patient groups. Furthermore, due to the retrospective nature of the evaluation, the relevant patient data were not available for all analyses, since, for example, laboratory values were not collected at the time of the interview with the depression questionnaire in all cases. This resulted in lower numbers of cases for certain sub analyses, which generally carries the risk of bias in the results. Missing data points in different variables also prevented us from using regression models for analysis instead of correlation as group size then became too small. In addition, as with all retrospective analyses, the risk of selection bias must be considered, because patients are preselected based solely on their presentation to a specialty outpatient clinic. Furthermore, the use of questionnaires entails the risk of response bias, particularly due to deliberate misrepresentation, self-representation, or influences from the interview situation.

Finally, it is important to keep in mind that due to the cross-sectional analysis, our findings can only indicate associations and point out trends but cannot prove causation.

## Conclusion

The present work demonstrates that depressive symptoms and depression are a significant and underdiagnosed comorbidity in children and adolescents with JIA. Pain, physical limitations, and poor treatment response (e.g., ineffective pain therapy, unsuccessful intensified therapy with biologics) seem to be crucial risk factors for depressiveness, which suggests that in addition to poor control of the somatic inflammatory disease burden, insufficiently effective therapy may also be associated with poor mental health. Consequently, we suggest for clinical practice that (1) regular screening for psychological comorbidities should be part of routine JIA consultations (2) JIA therapy should not be continued according to the current treat-to-target principles, but a therapeutic alternative should be sought until the desired therapeutic goal is achieved, and (3) when treating JIA, we need to take into account not only control of somatic symptoms, but also mental health when defining therapeutic goals.

## Data Availability

The data that support the findings of this study are not openly available due to reasons of sensitivity and are available from the corresponding author upon reasonable request.
